# Letter from E. Andrews, M.D. Ect.

**Published:** 1862-06

**Authors:** E. Andrews

**Affiliations:** Camp before Corinth


### ARTICLE XVIII.

TO THE MEDICAL EXAMINER.

CAMP BEFORE CORINTH, *May* 23, 1862.

MESSRS EDITORS:—I wrote you after the battle of Shiloh, but the uncertainties of the mail render all communications dubious. Presuming, however, that you received that letter, I will not repeat. The wounded were gradually shipped on steamboats, down the river; a large number stopping at Savannah. I learn that many secondary operations of resection and amputation became necessary there—part of them because the primary operations had not been performed which the cases required. Prof. H. A. JOHNSON stopped there not long since, and contributed his services for some time to their surgical care. After the departure of the wounded the attention of the army surgeons reverted to the sick. Camp diarrhœa is here as in all armies, the chief disease; and I will endeavor to give a more clear and careful account of it than is to be found in the books of Military Surgery. Almost all men and officers have an attack of it soon after entering the field service, and probably one-third of the army has it now. Only a moderate number, however, are incapacitated for duty. To call the disease a diarrhœa is, however, almost a misnomer, and conveys a very incomplete idea of its pathology. The alvine evacuations are but one symptom among many, and constitute but a small part of the disease. I have passed through the disorder myself and have closely observed its symptoms, both in myself and in hundreds of others. The conclusion which forces itself upon me, as well as upon other surgeons, is that nine-tenths of the sick of this army have one and the same pathological lesion, whether with or without diarrhœa, and that lesion is portal congestion. Its presence is manifested by a yellow or brown fur on the tongue; sometimes icterode skin and eyes; and almost always by deep red or yellow urine; scanty bile in the fæces; and a general tenderness of the abdomen. The ganglionic nervous system is probably affected, as both the sensations and the functions of every viscus below the diaphragm is thoroughly depraved. When the patient reaches this condition, one of two things occur: either the portal vessels partly relieve themselves by effusion into the intestines, in the form of diarrhœa or dysentery, or else, if the bowels withstand the pressure, a fever of bilious character sets in. If diarrhœa is the destined course of the symptoms, there is, coincident with the early portal congestion, a gentle looseness of the bowels for a few days, without loss of appetite or distress. About the fourth day, the irritative stage commences; the appetite fails; there are no pains in the stomach, bowels, or rectum. Often the patient vomits his food and medicine. In many instances, hemorrhoids present themselves and the diarrhœa or dysentery, as the case may be, is more frequent and profuse in its discharges. As manifested among us, the discharges from the bowels rarely become dangerous from their profuseness; hence, for the most part, we treat the cause first and regard the discharge as rather a temporary relief than an evil. In cholera time, of course all this would be changed, and the prompt suppression of the discharge would claim the earliest attention.

The treatment adopted is various; but as experience accumulates, most of the surgeons are compelled to recognize and act on the presence of the portal congestion by a free use of mercurials. I have tried opiates, astringents, cathartics, etc. abundantly, to avoid the use of what appears to a Chicagoan such a frightful quantity of colomel and blue mass, but in vain. I am obliged to confess they are necessary in this climate, and I have experienced their grateful effects in my own person. It is true, however, that other remedies have a temporary effect in arresting the diarrhœa. Thus, if you begin some day and give every one of twenty patients half-an-ounce of Epsom salts, as recommended by Tripler, you will find, the next morning, a large portion of them free from all evacuations. The same will be observed if you give them full doses of opium and acetate of lead, per chloride of iron, or any other potent astringents. It will be observed in these cases, however, that the patient is not relieved of his sense of illness; on the contrary, there springs up in a few hours a greatly increased severity of abdominal pain; frequently vomiting supervenes; the tongue remains foul; and great distress is apt to occur, and continue until the diarrhœa returns, when a partial sense of relief is felt. In the form of the disease, therefore, at present prevailing, we give but little attention to the diarrhœa directly—sometimes none at all—the patient protesting that his discharges relieve him. In sthenic cases I generally give two grains of calomel, with some prepared chalk and opium, giving a dose every two, three, or four hours, according to the severity of the case. In this way, the patients gain rapid relief, both from pain, feverishness, and diarrhœa. The malarious tendency, obvious in many cases, requires the addition of quinine.

The medical supplies of the army are improved, but still very imperfect. A surgeon can get only about half the articles required in the regulation supply table for field service. Perchloride and mur. tinct. of iron, so essential in erysipelas, cannot be had at all.

We had a smart skirmish in our division on the 15th instant, in which over 30 were wounded. In consultation with the surgeons, I proposed resection as a substitute for amputation, in a number of cases of shattered bone, such as heretofore have been condemned to the latter operation, and produced my chain saw to encourage the adoption of the plan. All favored it; and quite a furor for resection sprung up. We resected five cases: two of the middle of the shaft of the femur; two of the elbow; and one of the shoulder joint. One thigh, which had the femur shattered and the femoral artery cut, did not admit of the operation, but had to be amputated; making in all six capital operations. One of the patients with resected femur is dead; the amputated thigh will probably die also, being accompanied with great prostration. The remaining resections are doing well, and in the opinion of all here will subject the patients to much less risk than amputation. I was moved to this plan by the frightful mortality of amputations, being over 80 per cent. in cases of the thigh, and about 90 per cent. at the upper third, where half of them have to be made. Let no surgeon, however, think to escape the responsibility by splintering the limb and neglecting all operative assistance. Patients with the femur shattered by a bullet, almost all die, if not relieved by resection or amputation. Let every young military surgeon dissect the first limb amputated for gun-shot fracture which he sees, and he will see the cause of the danger. The bullet shatters the bone into fifty fragments, and drives them asunder into the surrounding flesh, almost as though a small shell had exploded in the centre of the limb. Large fragments of disorganized flesh and bone are therefore confined in the seat of injury. In a day or two, the bulging flesh closes the track of the bullet as with a tampon, so that not a particle of pus can escape. The foreign bodies produce enormous inflammation and suppuration. The pus prevented from escape burrows among all the muscles, and at length escapes at some distant point; meanwhile pain, the poison of rotting tissues, and the exhaustion of the discharges, are too much for human endurance. There is no doubt that amputation is better than this. What is claimed, however, by the advocates of resection is, that by laying the wound broadly open, and boldly removing all the fragments and sharp extremities of bone, and leaving a wide, free exit to the pus, the injury is converted into a simple and not extraordinarily dangerous wound, and that the frightful dangers of amputation are avoided. If a patient must be immediately carried a long distance in a wagon, the resected limb would be troublesome, but this is seldom absolutely necessary. In the superior extremity resection should *always* be preferred, unless the destruction of the limb is a matter of certainty.

There is constant skirmishing along our front, but nothing of great surgical interest has transpired. We are now in daily waiting for the great fight for Corinth, which may probably be over before my next letter. Yours truly,

E. ANDREWS.

